# Introduction to the Special Issue on Job Satisfaction in Fisheries in the Global South

**DOI:** 10.1007/s11205-012-0051-7

**Published:** 2012-05-16

**Authors:** Maarten Bavinck, Richard Pollnac, Iris Monnereau, Pierre Failler

**Affiliations:** 1 University of Amsterdam, Nieuwe Prinsengracht 130, 1018 VZ Amsterdam, The Netherlands; 2 University of Rhode Island, Kingston, RI USA; 3 Centre for Maritime Research (MARE), University of Amsterdam, Amsterdam, The Netherlands; 4 CEMARE, University of Portsmouth, Portsmouth, UK

## Opening Remarks

The job satisfaction of capture fishers is of more than sectoral interest. On a practical level the relevance is as follows: capture fishing is known to contribute in a major way to the degradation of the world’s oceans (Millenium Ecosystem Assessment 2005), and could possibly be relieved if fishers are induced to move out of fishing (Pauly et al. 1989). Whether fishers are actually inclined to do so or not, however, depends at least partially on their levels of job satisfaction. Comparative studies of job satisfaction—as attempted in this special issue—throw light on the extent to which fishers are attached to their work and are willing to give it up for alternative professions. More specifically, such studies provide evidence of labor conditions in a profession known to be exceptionally tough and even dangerous (ICSF 2003).

Academically too this special issue strives to make a contribution. Not only does it extend the reach of occupational studies of wellbeing and happiness to the unusual category of hunters and gatherers (Acheson 1981), but by attempting cross-cultural comparison, it is also methodologically innovative. The crucial question from this viewpoint is whether a single set of social indicators generates significant outcomes across various cultural and geographical settings (Bryman 2001).

## Fisheries and Job Satisfaction

The fisheries sector provides livelihood and employment to 45 million people worldwide (of whom 90 % live in developing countries) and is one of the world’s most important sources of animal protein, yet even the most impervious observers of world affairs will not have failed to notice that capture fisheries are in severe crisis (FAO 2010; World Bank 2009; Myers and Worm 2003; Pauly et al. 2003; Worm et al. 2006). Better fisheries governance is considered imperative (Kooiman et al. 2005; Constanza et al. 1998). For this purpose, scientists of various disciplines have developed assessment tools to establish the costs and benefits of various fisheries. Such assessments are carried out from the perspectives of ecology, economics, and the social sciences (Thorpe et al. 2011; Degnbol et al. 2006) and a multidisciplinary consilience approach (Failler and Pan 2007).

Social scientists study the human value of fisheries and the implications of fisheries decline in a comparative perspective. Job satisfaction studies are a valuable tool for this effort as they provide results that are comparable across different fisheries and geographical regions. As fisheries managers strive to reduce, or at least contain, the amount of fishing effort, and thereby increase the ecological sustainability of fisheries, the question to what extent fishers are attached to their profession is an important one (Cinner et al. 2009; Muallil et al. 2011). Previous studies suggest that fishing is often more than just another occupation, and that fishers have strong attachments to their work (Pollnac and Poggie 2008; Pollnac et al. 2001; Acheson 1981, 1988; Griffith and Valdes-Pizzini 2002; Glazier 2007; van Ginkel 2007; Smith and Clay 2010).

Fisheries are frequently divided into two or three types, depending on technology and capital- or labor-intensity (Johnson 2006). Although the industrial revolution that occurred in the twentieth century (Bavinck 2011; Platteau 1989) gave rise to large-scale fishing fleets that roam the oceans, the largest number of fishers still practice a style of small-scale, or artisanal, fishing that centers on the immediate surroundings of a landing center. An intermediate category of semi-industrial fishers has come up in between these poles. Data from the Food and Agriculture Organization point out that the absolute majority of contemporary fishers are based in developing nations of Asia, Africa and Latin America, whereas in so-called developed countries their numbers have been rapidly dwindling (FAO 2010). This special issue focuses on small-scale and semi-industrial fisheries in the developing world. These fishing types are known to include a large number of modes, or *métiers*, in which fishers, making use of innumerable gear types, target different species (Von Brandt 1984). Our assumption is that job satisfaction varies according to *métier.*

The International Labour Organization (ILO) has repeatedly drawn attention to the quality of labor conditions in fishing, and over time has drafted six Conventions and three Recommendations specific for the sector (ILO 2000; ICSF 2004, 2008; Bavinck and Chuenpagdee 2005). The regulations that have been drawn up, however, are known to apply more to large-scale than to small-scale fishing. The Food and Agriculture Organization thus concludes: “In practical terms, the scope of the existing labour standards in fishing, in general, does not include people who work on artisanal and small-scale fishing vessels.” (FAO 2004: 75). In line with this observation, none of the fisheries included in the present study have hitherto been subjected to ILO standards and are therefore largely self-regulated in terms of the quality of labor conditions.

### Job Satisfaction

Job satisfaction pertains to a subjective, individual-level feeling that reflects whether a person’s needs are or are not being met by a particular job (Lambert et al. 1999). It results from the worker’s comparison of actual outcomes with those that are expected, needed, wanted, or perceived to be fair or just (Lambert et al. 2001). Research on job satisfaction has demonstrated that it is a factor influencing the health of workers, both physical and psychological (Faragher et al. 2005; Pollnac et al. 2011). Job satisfaction is also considered to be a predecessor of turnover intent of workers (Lambert et al. 2001), and therefore highly relevant for the field of human resource management (Cranny et al. 1992).

Investigations of job satisfaction commenced in the 1930s (Locke 1969, Bruk-Lee et al. 2009) but increased in the 1960s and 1970s with the development of labor studies (Marshall 1994: 707). Maslow (1954)—who suggested that human needs form a five-level hierarchy ranging from physical needs, safety, belongingness and love, esteem to self-actualization—was particularly influential in this effort (Lu et al. 2005). Following a Maslowian line of thought, job satisfaction became approached from the perspective of need fulfillment. In more recent years an attitudinal perspective has been added to the study of job satisfaction (Spector 1997).

Some of the main discussions in the job satisfaction literature concern determinants. Locke (1969) has argued that the origins of job satisfaction could be located: (1) either exclusively in the job, (2) exclusively in the worker’s mind, or (3) as a consequence of an interaction between the worker and his work environment. Nearly three decades later Spector (1997:30) distinguishes two categories of antecedents: individual factors and factors related to the nature of the job and its environment.

The first category of studies investigates the relation between personality traits and job satisfaction (Bruk-Lee et al. 2009). Research comparing identical twins show evidence that genetic factors influence variance in work attitudes by as much as 30 % (Arvey et al. 1989). Pollnac and Poggie (2006, 2008) have thus argued that individuals with a personality type that can be characterized as active, adventurous, aggressive, and courageous seek out activities (including work) that satisfy these needs. The fishing occupation is one of these.

The second category studies the nature of the job, its environment and job satisfaction. Economists have focused on a range of related issues, such as how relative income or union membership (Bender and Sloane 1998; Meng 1990) and the role of gender affect job satisfaction (Smyth et al. 2009; Clark 1997). There is thus a growing literature on what makes a good job and how the attributes that employees seek for, impact job satisfaction as ‘satisfied employees tend to be more productive, creative and committed to their employers’ (Syptak et al. 1999).

Scholars have generated a variety of tools for assessing job satisfaction, which allow for adaptation to specific purposes and work fields. The job satisfaction studies that were undertaken in fisheries have largely been based on Maslow’s (1954) hierarchy of needs (Bavinck and Monnereau 2007), distinguishing three basic categories: views on the fulfillment of basic needs, social needs, and needs of self-actualization. In order to measure the results hereof in fisheries, Pollnac and Poggie (1988) designed and tested a list of 22 items, with two additional questions on overall job satisfaction. The first additional question asks whether a fisher would still go into fishing if he had his life to live over again; the second whether or not he would advise a young man to go into fishing. Factor analyses of the 22 items resulted in factors reflecting Maslow’s hierarchy of needs stimulating further research on the items. Other scholars made small modifications to this original set of items yet their analyses indicated that it maintained its overall structure (Gatewood and McCay 1988, 1990; Binkley 1995). Comparing these approaches, one of the significant findings has been that nonmonetary aspects constituted important components in determining job satisfaction.

In line with global job satisfaction studies, job satisfaction studies in fisheries have been plentiful, but mostly based in North America (see Pollnac and Poggie 1988; Pollnac et al. 2006; Smith 1981; Apostle et al. 1985; Gatewood and McCay 1988, 1990). This regional bias has generated a corpus of studies on human populations that possess a large measure of cultural homogeneity. Moreover, the fisheries that were included are more-or-less industrialized and integrated into the world economy, to the neglect of smaller-scale fisheries in different cultural settings. Slowly job satisfaction studies in other regions have been gaining ground (Pollnac et al. 2001; Monnereau et al. 2010; Pollnac et al. 2011). An international and inter-métier comparison of job satisfaction in fisheries has, however, not been carried out, and the present volume constitutes a unique test of the methodology.

### Methodology for the Present Study

This special issue is rooted in an interdisciplinary research project with the acronym ECOST, which was funded by the European Commission FP6 program in the period 2005–2010.^1^ The aim of the ECOST project was to assess the societal costs (ecological, economic and social) of fishing activities and policies in three regions (the Caribbean, West Africa and South and East Asia) in order to contribute to a better management of aquatic resources.

The ECOST project included a selection of social scientists from these areas as well as from Europe, who investigated job satisfaction among different fishing métiers. For the purpose of comparison, the members of the team, who were later joined by Richard Pollnac from the University of Rhode Island, first reviewed and amended the job satisfaction assessment tool, which was developed for North American purposes. A revised version of the tool was subsequently tested on a selection of fishing métiers that are of importance in the countries concerned. This special issue reports on the studies carried out: three in Asia (India, Thailand, and Vietnam), two in West Africa (Senegal and Guinea Bissau) and four in the Caribbean (Nicaragua, Dominican Republic, Jamaica and Belize).

The social scientists involved in ECOST reviewed the Pollnac and Poggie (1988) list of 22 items of job satisfaction in a preparatory workshop in Amsterdam (1–3 Nov. 2006). The objective was twofold: (a) to adapt the list of indicators to more adequately reflect the concerns of fishers in developing country settings, and (b) to include issues of current concern, such as management and resource depletion. As a result of this consensual review, the list expanded to 27 items, organized in 5 categories plus an additional three yes/no questions in a sixth category. All but four of the original indicators designed by Pollnac and Poggie (1988) were included in the new list, for purposes of comparison with North American findings^2^ (see Table 1).

**Table 1:** 

Category 1: Basic needs
addition of food security concern (question 10 regarding the ability to feed your family) and level of catches (question 11)
Removal of item ‘doing deckwork on vessel’
Category 2: Social needs
Removal of two items ‘ability to come and go as you please’ and ‘peace of mind’
Category 3: Self-actualization
Removal of item ‘working outdoors’
Category 4: Management
Pollnac and Poggie (1988) included only one question on the performance of state and federal officials. Five questions on conflict and conflict resolution, rules and regulations, possibilities for participation, and overall management were added
Category 5: Nature
This is an entirely new category of items with only two questions: views on the condition of the landing place, and views on the condition of fish stocks
Category 6: General questions
One of Pollnac and Poggie’s (1988) general questions, on whether the respondent would advise a young person to enter fishing, was maintained. To this were added two questions regarding the wish to move from one into another fishing métier, or to shift to a job outside fishing

In comparison with the original Pollnac and Poggie (1988) list, the differences are as follows:

Upon completion of the English language survey (see Appendix), the researchers translated the list into various local languages. Each social scientist subsequently conducted a representative sample of surveys among fishers participating in a fishing métier, also distinguishing between positions such as skipper or crew member.

Papers on the basis of these initial results were first presented and discussed at the *MARE People and the Sea: Who owns the coast?* conference (Amsterdam 2007). Additional field research was subsequently carried out to validate earlier findings.

## This Special Issue

The seven papers that make up this special issue are organized geographically. The first two papers refer to small-scale fisheries in West Africa, with cases in Guinea Bissau and Senegal. In both cases the people involved are small-scale fishers. This region boasts a rich marine ecology and a large and growing fishing population. Important issues include the fishing agreements which have been concluded with the European Union, the implementation of Marine Protected Areas (MPA), and the existence of a large Illegal, Unregulated and Unreported (IUU) industrial fishery, both of which are believed to impinge on the opportunities of small-scale fishers.

The next three papers relate to important fisheries in South and South-East Asia, in the countries of India, Vietnam, and Thailand. FAO (2010) notes that the largest concentration of fishers in the world lives in Asia (85 %), and that this population is steadily increasing. At the same time there are concerns about the longer term sustainability of the fisheries. The fisheries studied in Vietnam and India are semi-industrial shrimp trawl fisheries. In Thailand, however, the fishers studied employ a métier consisting of a variety of small-scale fishing gears.

The final set of papers discusses job satisfaction in the mosaic of nations that comprise the Wider Caribbean. One paper has a specific country setting (the Dominican Republic) while the second paper makes a comparison between three countries (Belize, Jamaica and Nicaragua) with regard to the important lobster fisheries. Although the Caribbean does not host any of the world’s major fisheries, the sector is often important for local employment and food security. The fishery of the Dominican Republic is of a small-scale using a variety of gears. In Nicaragua and Jamaica an industrial fishery also exists, but the sample of fishers in this paper is restricted to small-scale lobster fishers.

The special issue is therefore comprised of a mixture of small-scale and semi-industrial fisheries using a variety of gears and targeting a variety of species. The concluding paper aims to provide a comparative analysis of the various country data and the perspectives on job satisfaction studies in the future. Taken together, the articles in this special issue provide a comprehensive framework for the validation of job satisfaction research, complement earlier research on job satisfaction carried out in the North, and add to current research being undertaken in development studies on wellbeing. In addition, it aims to introduce novel ideas to the agenda of job satisfaction in fisheries. We hope this special issue serves as a useful resource for researchers and users of the job satisfaction indicators and of other large-scale assessment social indicator projects pertaining to the wellbeing of fishers. In particular, we hope that the ideas and findings presented in this issue contribute the practices and decisions made by managers and decision makers, namely, to advance fishers’ satisfaction with their job, and consequently, the wellbeing of their families, and communities. Additionally, an understanding of job satisfaction among fishers will assist in developing management plans that can provide for appropriate alternative occupations for fishers displaced by necessary reductions in effort.

## Footnotes

1 The full title of the project (nr 003711) is *Ecosystems, Societies, Consilience and the Precautionary Principle: Development of an Assessment Method to Establish the Societal Cost of Best Fishing Practices and Efficient Public Policies* (see www.ecostproject.org). ECOST was coordinated by the University of Portsmouth (UK) and involved 22 partners across three geographical regions.

2 Pollnac et al. (2011) found striking commonalities between an analysis of the same job satisfaction attributes in New England, Alaska and several Caribbean nations.
